# Becoming Stressed: Does the Age Matter? Reviewing the Neurobiological and Socio-Affective Effects of Stress throughout the Lifespan

**DOI:** 10.3390/ijms21165819

**Published:** 2020-08-13

**Authors:** Aroa Mañas-Ojeda, Francisco Ros-Bernal, Francisco E. Olucha-Bordonau, Esther Castillo-Gómez

**Affiliations:** 1 Department of Medicine, School of Medical Sciences, Universitat Jaume I, 12071 Castellón de la Plana, Spain; al384380@uji.es (A.M.-O.); fros@uji.es (F.R.-B.); folucha@uji.es (F.E.O.-B.); 2 Spanish National Network for Research in Mental Health, Centro de Investigación Biomédica en Red de Salud Mental (CIBERSAM), Planta 0, 28029 Madrid, Spain

**Keywords:** stress, amygdala, early life, adult, old age, neuronal plasticity, endocannabinoid, anxiety, depression, sociability

## Abstract

Social and affective relations occur at every stage of our lives. Impairments in the quality of this “social world” can be exceptionally detrimental and lead to psychopathology or pathological behavior, including schizophrenia, autism spectrum disorder, affective disorders, social phobia or violence, among other things. Exposure to highly stressful or traumatic events, depending on the stage of life in which stress exposure occurs, could severely affect limbic structures, including the amygdala, and lead to alterations in social and affective behaviors. This review summarizes recent findings from stress research and provides an overview of its age-dependent effects on the structure and function of the amygdala, which includes molecular and cellular changes, and how they can trigger deviant social and affective behaviors. It is important to highlight that discoveries in this field may represent a breakthrough both for medical science and for society, as they may help in the development of new therapeutic approaches and prevention strategies in neuropsychiatric disorders and pathological behaviors.

## 1. Introduction

Human beings are a highly social species. This does not make us special, since all mammals exhibit some degree of social behavior, such as cooperation, affiliation or aggression, that allows us to survive and thrive. However, socio-affective relations are so important for human health and well-being that many psychiatric and neurological disorders are characterized by prominent impairments in social or affective functioning. Autism spectrum disorder (ASD), schizophrenia, bipolar disorder, major depression and social anxiety disorder are just some examples [1,2,3].

Social and affective behaviors occur at every stage of our lives, beginning in infancy with caregiver attachment, followed by peer interactions during childhood and adolescence and the formation of pair-bonds and paternal behaviors during adulthood and old age [4]. Our “social world”, that is, the environment and people around us, has a crucial role in the development and maintenance of our socio-affective behavior. While normative or positive stimuli are necessary for proper neurological and behavioral development, impairments in the quality of this “social world” can be exceptionally detrimental and lead to psychopathology [2,5].

The mammalian brain is extraordinarily plastic, capable of restructuring synaptic connections in response to a changing environment. In fact, during brain development, there are stages of heightened plasticity, the so-called critical periods or critical windows of plasticity, when environment is extremely effective in producing lifelong changes in the brain [6]. These critical periods are a time not only of opportunity, but also of great vulnerability. Exposure to supportive or enriching situations during these periods may favor the long-term remodeling of neurobehavioral trajectories so as to promote better stress-coping strategies [7]. Conversely, the exposure to stressful or traumatic situations during critical periods of plasticity may impair these trajectories and therefore contribute to the emergence of psychopathologies and abnormal behaviors later in life (affective disorders, schizophrenia, violence, addiction, etc.) [5,8,9]. Among the different age stages at which individuals are more vulnerable to the effects of stress, the majority of studies in animal models have focused on the early postnatal period [7]. Lately, the peri-pubertal period (which encompasses late childhood and adolescence) has also attracted significant attention. In fact, current evidence has identified this age stage as a period of enhanced vulnerability to the effects of stress, and also as a strong modulator of socio-affective behaviors in adulthood [5,10]. Moreover, many neuropsychiatric disorders are thought to emerge during this period [11].

The ability to cope with a stressor depends on several intrinsic factors, including age, as was just mentioned, but also on some properties of the stressor itself, such as duration, strength and unpredictability [12,13]. In fact, the response to an acutely stressful stimulus is considered to be beneficial, since it is an evolutionary mechanism of adaption to an environmental change. However, if a stressor becomes chronic, or if it is unpredictable or too severe, the ability to cope with the stressor can be impaired, leading to behavioral alterations or even to the emergence of psychopathologies later in life [13,14]. This is the so-called maladaptive response to stress [14] and will be the focus of the present review. This maladaptive response to stress, depending on the stage of life in which stress exposure occurs, could differently affect social and affective behavior [10,14,15]. These effects can be persistent or reversible, and can be suffered just after stress exposure, later in life, or can even affect several offspring generations, in what is known as the transgenerational inheritance of stress [10,16,17,18,19].

Violence, particularly against women and children, is one of the most important social and public health problems worldwide, affecting up to 1 billion children [20] and nearly one-third of the world’s female population [21]. Many stressors have been associated with these high levels of violence, including social neglect [22] and socio-economic inequality [23]. When children are raised in disadvantaged social environments, they are at risk of developing severe mental health problems during adulthood, including anti-social and violent behaviors. These problems can even be inherited by future generations, as was mentioned above [24]. Social neglect is one of the important factors that can elicit this pathological aggression, especially when it occurs in early life [5,10,24], although the underlying neural bases of such phenomenon remain poorly understood.

**Figure 1:**
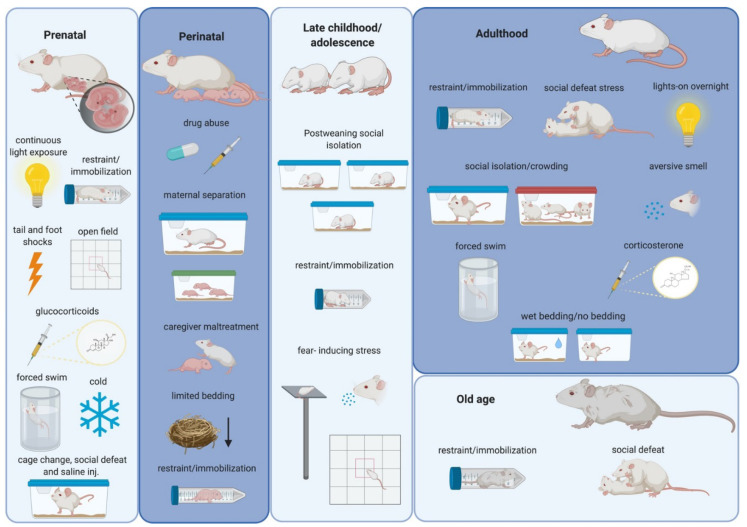
Schematic drawing summarizing the most frequently used stressors in rodents during different periods of the lifespan. Alterations in the structure and function of the amygdala and in social and affective behaviors have been related to these stressors (see text). Created with BioRender.com.

In this regard, experimental animal models devoid of human cultural connotations constitute a valuable tool for studying anti-social behaviors and affective impairments related to stress. Animal models are also indispensable for investigating the molecular and structural changes in the brain related to these stressors (Figure 1). In addition, as numerous parallelisms have been identified between rodents and humans regarding age periods of heightened susceptibility to environmental stimulation, studies using rodent models of stress can complement studies in humans [7].

Among all the regions that have been implicated in social and affective behavior, the amygdala seems to play a pivotal role [25]. Glucocorticoid receptors are highly expressed by neurons in the amygdala, what makes these neurons one of the main targets of the neuroendocrine mediator of stress, the hypothalamic pituitary adrenocortical (HPA) axis [26]. Research in humans and animal models has shown that the amygdala is a critical mediator of social behavior and affective processing [25,27,28]. In fact, alterations in this brain region have been found in many neuropsychiatric and behavioral disorders, including schizophrenia, ASD, major depression and social phobia, among others [2,25,27,28]. These studies in humans and animal models, together with classic studies based on amygdaloid lesions in nonhuman primates, have positioned the amygdala at the center of the socio-affective brain [27]. Excitatory and inhibitory amino acids, glucocorticoids and a growing list of intra- and extracellular mediators, which includes endocannabinoids (eCB) and brain-derived neurotrophic factor (BDNF), have also been identified to play key roles in social and affective processes [29,30]. Enviromental insults, including stressful events, result in an incessantly changing pattern in the expression of genes related to these aminoacids, glucocorticoids, eCB and BDNF. These changes may be mediated by epigenetic mechanisms, as we will also discuss.

This review summarizes the recent findings of stress research and provides an overview of its age-dependent effects on the structure and function of the amygdala, which includes molecular and cellular changes, and how they can trigger deviant social and affective behaviors. The findings in this field may represent an advance both for medical science and for society, as they may help in the development of new therapeutic approaches and prevention strategies in neuropsychiatric disorders and pathological behaviors.

## 2. Stress Exposure during the Prenatal Period: Prenatal Stress

The prenatal period is defined as the developmental period between conception and birth. Exposure to stress during this period influences fetal brain development [31] and raises the risk of the offspring developing mental diseases [32,33]. Before we start reviewing findings in prenatal stress research, it is important to clarify terminology. Prenatal stress refers to the stress suffered by mothers during the gestational period that affects the offspring [31]. In humans, it has been demonstrated that prenatal exposure to unpredictable or chronic stressors, such as adverse life events, natural disasters or social pressures [34,35], increases the risk of suffering several psychopathologies, including schizophrenia [36,37,38], ASD [39,40], and major depression- and anxiety-related disorders [41]. Importantly, recent studies have confirmed what has long been theorized, but not yet observed in humans: that stress suffered during pregnancy is reflected in the connectional characteristics of the offspring’s brain, independent of the influences of the postnatal environment [42]. Decreased amygdala connectivity has been described in the neonates of mothers exposed to gestational stress, with additive effects associated with preterm birth. Specifically, extremely preterm neonates (<28 weeks gestation) with prenatal stress exposure showed decreased amygdala connectivity with the thalamus, the hypothalamus, the brainstem, the fusiform and the insula, when compared to extremely preterm neonates without prenatal stress exposure, very preterm neonates (<32 weeks gestation) or term controls (37–41 weeks gestation). Comparison among groups showed that term controls had the higher amygdala connectivity, and preterm neonates with prenatal stress exposure had the lower connectivity [42,43].

Rodent models of prenatal stress (Figure 1 and Table 1) are mainly based on the exposure of the fetus to glucocorticoids via the placenta, either by the administration of synthetic glucocorticoids to the pregnant female or by the exposure of the mother to stressors during the gestational period [13]. Interestingly, many studies in rodents have demonstrated that different maternal stressors can replicate some of the behavioral abnormalities observed in the offspring of women subjected to gestational stress [34].

**Figure 2:**
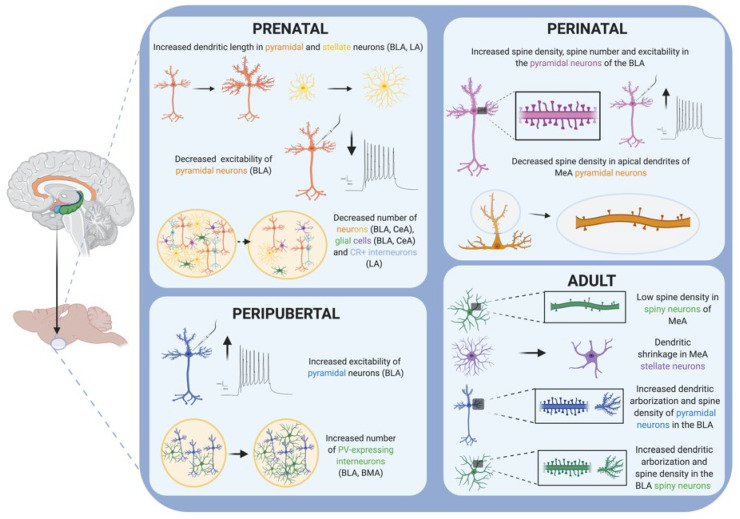
Schematic drawing summarizing the main cellular alterations reported in the amygdala of rodents after stress exposure in different periods of the lifespan. Created with BioRender.com.

Restraint or immobilization, with or without bright light, is still the most popular stressor used in pregnant rodents [34]. The pyramidal and stellate neurons from the basolateral (BLA) and lateral (LA) nuclei of the amygdala of adult male mice (8 week-old) that were exposed to restraint stress during the perinatal period (from gestational day 14 until parturition) showed increased dendritic length [44] (Figure 2). Interestingly, when perinatal restraint stress was combined with maternal exercise (from gestational day 1 to 17), these morphologic changes observed in the amygdala of adult mice were blocked [44]. Restraint stress suffered during gestation also led to decreased serotonergic metabolism and increased corticosterone (CORT) response to social interaction in the adult offspring [45].

Stress induced by continuous light exposure is considered to be a potent circadian rhythm disruptor, leading to abnormal behavior in adult rodents related to decreased circulating melatonin levels [47]. In the same way, as the development of the circadian clock begins in the prenatal period, continuous light exposure during gestation decreased mobility and exploratory activity in the offspring, and up-regulated circadian-related gene RORA in the amygdala [47]. Interestingly, major depression and ASD have also been related to altered circulating melatonin levels in humans [56,57].

Many studies have also reported abnormalities in socio-affective behavior and amygdala function in the adult offspring of female rats exposed to chronic unpredictable stress during gestation [50,51,58]. Specifically, decreased sociability and anxiety levels [50], together with reduced neuronal and glial number in the BLA and central (CeA) nuclei of the amygdala [51], as well as the reduced excitability of BLA principal neurons [50], have all been observed in the offspring. Other studies have reported increased oxytocin receptor (OXTR) binding in the CeA, and decreased social interaction without changes in anxiety-related behavior in adult offspring subjected to prenatal unpredictable stress [52], concluding that this model could be an appropriate animal model for some aspects of schizophrenia social withdrawal, since exposure to this stressor degrades social interaction behaviors [52]. Surprisingly, fluoxetine [59], but not escitalopram antidepressant treatment [58], has been shown to block the effects of prenatal stress on socio-affective behavior and amygdala function, when administered prenatally. These results suggest a role for serotonin in the socio-affective effects of unpredictable prenatal stress. In this line, unpredictable stress suffered by pregnant 5-HTT heterozygous mice (low activity serotonin transporter) results in the offspring showing ASD-like behavioral characteristics, including decreased social interaction and social interest [48].

Glucocorticoid exogenous administration during the prenatal period also leads to affective and social deficits in adulthood, including depressive-like behavior [53] and impairments in social interaction [54]. Dexamethasone treatment during late gestation reduced the density of calretinin-expressing cells in the LA nucleus of the amygdala of adult rat offspring, but no differences were observed in the BLA nucleus [55].

Among all neurotrophins, BDNF stands out for its high level of expression in the brain and its potent effects on synaptic plasticity, playing a critical role during neuronal development. The offspring of pregnant mice subjected to 8 days of unpredictable stress have shown decreased BDNF expression in the amygdala, together with an increased expression of DNA methyltransferases 1 and 3a (DNMT1, DNMT3A) and reduced methylation in *Bdnf* exon IV in the amygdala, both at weaning and in adulthood [49]. These results point to epigenetic mechanisms mediating the prenatal stress-induced reduction in BDNF expression in the amygdala [49]. Some studies have correlated this downregulation of BDNF expression with schizophrenia and depression-like symptoms in mice and humans [34,49]. Interestingly, a recent human study found that not only gestational stress but also the history of trauma suffered by the mother was associated with epigenetic regulation of Bdnf in their newborns (Bdnf methylation and BDNF protein expression measured from umbilical cord blood after birth). Male neonates showed augmented BDNF expression when mothers were exposed to child abuse, and elevated Bdnf methylation when mothers experienced fear. By contrast, female neonates just showed decreased BDNF expression in correlation with maternal fear [60].

**Figure 3:**
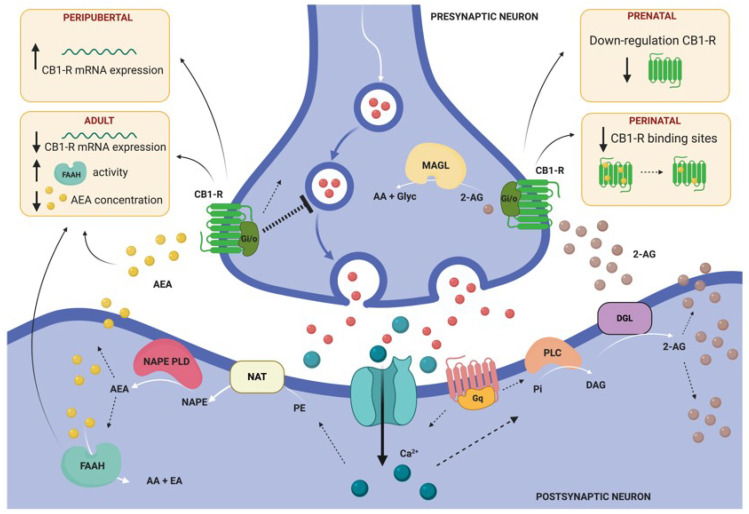
General model illustrating retrograde endocannabinoid (eCB) signaling and summarizing the main findings of altered eCB system in the amygdala of rodents subjected to stress in different periods of the lifespan. This model illustrates the two primary biosynthetic pathways for anandamide (AEA) and 2-arachidonoyl glycerol (2-AG). AEA and 2-AG migrate from postsynaptic neurons to bind presynaptic-located cannabinoid type 1 receptors (CB1-R). Once activated, CB1-R couple through Gi/o proteins to inhibit adenylyl cyclase and regulate ion channels, and ultimately suppress neurotransmitter release. Endocannabinoid signaling is then terminated by degrading enzymes. AEA is mainly hydrolyzed to arachidonic acid (AA) and ethanolamine (EA) by fatty acid amide hydrolase (FAAH), located post-synaptically [63]. 2-AEG is mainly hydrolyzed pre-synaptically to AA and glycerol (Glyc) by monoacylglycerol lipase (MAGL). Abbreviations: DAG (diacylglycerol), DGL (DAG lipase), NAPE (N-arachidonoyl-PE), NAPE-PLD (NAPE-phospholipase D), NAT (N-acetyltransferase), PE (phosphatidylethanolamine), Pi (phosphatidylinositol), PLC (phospholipase C). Illustration created with BioRender.com.

It is well known that exposure to abuse drugs during the prenatal period can have long-lasting neurobehavioral consequences, including alterations in the offspring’s stress response, in part due to alterations in the eCB system of the fetus [61]. Interestingly, several studies have demonstrated that stress exposure can have a similar impact on the eCB system to that described for prenatal exposure to abuse drugs. For instance, prenatal restraint stress combined with tail shocks have been shown to downregulate the expression of cannabinoid type 1 receptors (CB1-R) in rodent offspring [46,62]. Remarkably, as we will discuss in the following sections, the eCB-dependent response to stress is determined by the age at which stress is experienced (Figure 3).

## 3. Stress Exposure during the Perinatal Period: Perinatal Stress

The perinatal period is defined as the period immediately before and after birth. In humans, it lasts until the baby is 28 days old, and in rodent models until weaning age [64]. Alterations in the interaction between parents and their progeny during this period are a very important cause of stress and may induce neuroanatomical and behavioral impairments in the progeny that can become evident either immediately or later in life [13]. The great majority of rodent models of perinatal stress (Figure 1 and Table 2) are based on disturbed maternal care and are typically performed either by inducing behavioral alterations in the mother (therefore affecting the quality of interactions with the pup) or artificially disrupting this maternal care (maternal separation) [13]. In several studies, maternal separation has been combined with the exposure to short-term stressors (10 min), such as noise, bright lights, low temperature, pain and handling, with the aim of modeling in rodents the stress of human neonates in the intensive care unit [13,65].

It has been demonstrated that bad caregiving conditions (i.e., limited bedding) during the pre-weaning period induces sexually dimorphic alterations in the BLA neuronal structure. Specifically, this decreased quality of maternal care has been associated with dendritic hypertrophy (increased dendritic length), increased spine density and enhanced excitability in the BLA of male but not female rodents at weaning age [66] (Figure 2). Male pups also showed a tendency towards an increase in BLA volume. Importantly, these changes have also been related to increased anxiety-like behavior and decreased social contact during adulthood, but only in male rats. All these results suggest that either females are more resistant to the stress of bad caregiving conditions, or that there are compensatory mechanisms activated only in females that prevent the morphological consequences of the BLA hyperexcitability that occurs in the pre-weaning period [66]. These findings are very interesting because they are in contrast with the fact that female pups received more adverse care compared to their male littermates, and this can explain why female pups manifested more behavioral and epigenetic consequences [78]. Remarkably, although limited bedding is a more naturalistic paradigm, and one might think that this stressor may induce less behavioral and neuroanatomical effects than those induced by a more controlled stressor, in a study comparing the effects of limited bedding with olfactory classical conditioning in pups, researchers found that both models induced the same deficits in social behavior and depressive-like symptoms during adolescence, and that those deficits were equally correlated with an increase in amygdala neuronal activity (c-Fos expression) [70].

DNA methylation is an epigenetic mechanism that explains how early-life experiences can alter the behavior and lead to disease development [67]. Rats that were continually exposed to caregiver maltreatment outside the home cage during their first 7 days of life showed increased methylation of the *Bdnf* gene in the amygdala, during both infancy and adulthood [68]. Other studies in rats demonstrated that the effects of perinatal caregiving maltreatment are also detectable at the level of the epigenome in the amygdala of adolescent animals, in a sexually-dimorphic way [69]. Specifically, adolescent females showed increased methylation levels of exon IV *Bdnf* DNA, but no significant alterations in 5-mC (methylation) or 5-hmC (hydroximethylation) global levels. By contrast, adolescent males did not show alterations in *Bdnf* DNA methylation levels but showed decreased 5-hmC levels in the amygdala. [69]. In a more naturalistic paradigm, where the mother rat maltreats the pups in the home cage, it has been demonstrated that social behavior deficits and amygdala dysfunction (decreased volume, neurogenesis, c-Fos reactivity and local field potential) related to this stress require both an increase in the stress hormone corticosterone and the context of maternal presence [70,79]

Early cannabinoid exposure leads to socio-affective dysfunction and alterations in cannabinoid and opioid transmission [80]. For example, prenatal and perinatal Δ9-tetrahydrocannabinol (Δ9-THC) administration decreased the density of µ-opioid receptors in the posteromedial cortical amygdala in adult rat males, while in adult females, contrary results were found [77]. However, as also happens in the prenatal period, there could be alterations in the eCB system at the perinatal age without exposing animals to drugs of abuse. For instance, mice exposed to maternal separation showed decreased CB1-R binding site densities within the amygdala, in both adolescence and adulthood. By contrast, alterations in the concentration of AEA and 2-AEG were only documented in infant rats after maternal separation stress [71,72] (Figure 3).

Maternal separation stress has also been related to decreased anxiety in male offspring during infancy and adulthood, and persistent increases in BDNF and TrkB protein levels in the CeA of adult males [73,74]. Decreased social interaction with unknown conspecifics was also observed in adult and adolescent rats subjected to a maternal separation stress protocol [75]. This behavioral impairment was correlated with a reduced expression of BDNF in the amygdala of adolescent and adult rats. By contrast, changes in the level of DNA methyltransferases (*Dnmt3a*, *Tet3*) were only found in the amygdala of adolescent rats [75]. Neuronal structure has also been demonstrated to be affected by maternal separation stress (Figure 2). Specifically, decreased spine density in the apical dendrites of the pyramidal neurons of the medial nucleus amygdala (MeA) was observed in peri-puberty rodents (3-week-old *Ortodon degus*) that were subjected to perinatal stress induced by maternal separation [76].

## 4. Stress Exposure during Late Childhood and Adolescence: Peripubertal Stress

Puberty is defined as “the peak phase of maturation of the hypothalamo–pituitary–gonadal axis, when alterations in gonadotropin levels in circulation and elevated levels of sex steroids occur” [64]. The peripubertal period (or periadolescent period) is characterized by hormonal and neurophysiological changes that make peri-adolescent individuals unique in their species compared to younger or older members [5,81]. In humans, this period includes late childhood and adolescence, and is broadly considered to range from 10–12 to 18–19 years of age [5,81,82]. In rodents, the first observable signs of puberty have been reported around postnatal day 28 in mice [64] and postnatal day 41 in rats [83], so the peripubertal period can considered by researchers to range from the age of weaning (postnatal day 21) to late adolescence (7–8 weeks of age) [5,64,81].

In humans, substantial evidence indicates that exposure to highly stressful or traumatic events during late childhood and adolescence is a predisposing factor for developing psychopathologies and alterations in socio-affective behavior, including schizophrenia [84,85,86], major- depression and anxiety-related disorders [87,88], violence [89,90,91,92] and impaired social function [84,85,93].

Stress susceptibility differs between the peri-pubertal and the adult brain in both humans and rodents. In fact, although the glucocorticoids levels in peri-adolescent individuals are similar to those of individuals at other age stages, when exposed to stress, both the duration and the amount of glucocorticoids are higher during puberty. This points to puberty as another critical period for shaping the HPA axis’ responsiveness [13]. The impact of stress in rodents also differs behaviorally between peri-puberty and adulthood (i.e., adolescent female rats exhibit “play and avoidant behaviors”, but not aggressive behaviors when facing a resident female, and exhibit less anxiety in response to social defeat stress). By contrast, the programming effects of stress during puberty are very similar to those observed in earlier age stages, thus adolescent rats exposed to stress show altered socio-affective behaviors in adulthood [5,13].

Many different rodent models of peripubertal stress have been described (Figure 1 and Table 3) in an attempt to best mimic the effects of stress suffered during late childhood and adolescence in humans.

The post-weaning social isolation stress model (PWSI) is one of the most widely used rodent models of social neglect [24]. In most laboratories, PWSI protocol consists of the housing of pups in individual cages from the first day of weaning (postnatal day 21) until adulthood, a time period covering the end of childhood and all adolescence [8,9,94,95,96,97]. Isolated animals are usually reared in the same room as other group-housed or isolated-reared rodents, so they have auditory, olfactory and sometimes visual but not physical contact with other conspecifics [96,97]. Normal social and emotional development needs physical interactions with conspecifics from birth to early adulthood, thus it is not surprising that PWSI leads to alterations in social and emotional behavior, including pathological aggression [8,9], deficient social communication [9] and increased anxiety [95,97]. Interestingly, re-socialization, a laboratory model for behavioral therapy, fails to correct the PWSI-induced pathological aggression, suggesting that this stressor induces long-lasting changes in socio-affective behavior [94]. PWSI also induced a permanent decrease in BDNF expression in the amygdala, which was also demonstrated to be re-socialization-resistant [94]. Adult rodents subjected to PWSI have larger amygdala volumes, specifically in the BMA, BLA and CeA nuclei [97], and increased BLA pyramidal cell excitability, measured by means of electrophysiology [95]. Regarding the molecular and cellular effects of PWSI in the amygdala of adult rodents, many findings have been described, especially regarding the structure and plasticity of inhibitory networks [96,97]. Rats subjected to PWSI showed increased GAD67 protein levels in the centromedial (CeM), MeA and BLA nuclei during adulthood [96]. Moreover, an increased number of parvalbumin-expressing interneurons was found in the BLA and BMA nuclei of PWSI-mice [97]. In the whole amygdala, a decreased expression of the PSA-NCAM, a plasticity-related molecule, was found, and in the La and BLA nuclei a decreased VGLUT1/VGAT ratio was reported [97]. In the same study, the level of mRNA encoding CB1-R was found to be increased in the amygdala of PWSI-mice [97], highlighting the influence of peripubertal stress on the eCB system (Figure 3).

Exposing rats to unpredictable stress by using fear-inducing stressors (open field, fox-odor, elevated platform) during the peripubertal period has been demonstrated to induce changes in several behavioral domains during adulthood, including pathological aggression, sociability deficits, and increased anxiety and novelty reactivity [83,98,99,100,101]. Moreover, hyperactivity in the amygdala has been observed in parallel with all these behavioral alterations [98]. Peri-pubertal unpredictable stress also led to molecular changes in different nuclei of the amygdala [83,100,101]. Specifically, peri-pubertally stressed rats showed increased mRNA levels of the *N-methyl D-aspartate receptor subunit 1 (NR1)*, reduced mRNA levels of the *glutamic acid decarboxylase 67 enzyme (GAD67)* and a heightened excitation/inhibition ratio (measured as the ratio between vesicular glutamate transporter 1 (VGLUT1) and vesicular GABA transporter (VGAT)) in the CeA nucleus of the amygdala [83]. An increased mRNA expression of the glucocorticoid receptor (GR) and a decreased number of GR-expressing cells in the CeA was also observed [101]. In the LA, BLA, BMA, MeA and CeA nuclei of the amygdala, reduced GAD67 and GABA-A receptor α3 was also reported [100]. Importantly, Dr. Sandi’s group also demonstrated that the peripubertal stress protocol they applied in all their studies [83,98,99,100,101] must be applied to its full extent in order to replicate the behavioral and neurobiological alterations they described, since the same protocol applied during the juvenile period only, or during the pubertal period, was demonstrated to be insufficient to produce the same effects [83].

As we have pointed out above, GABAergic inhibition is a key regulator of the activity of the amygdala, including the LA and BLA nuclei, and it has been demonstrated to have a critical influence over the behavioral and emotional sequelae resulting from stress [102,103]. Rats subjected to repeated restraint stress showed reduced GABAergic inhibition of the La projection neurons associated with increased anxiety [102]. When tail shock was added to the restraint stress protocol, animals also showed a severe impairment in the serotoninergic modulation of GABAergic transmission in the BLA, associated with amygdala hyperexcitability [103].

## 5. Stress Exposure during Adulthood: Adult Stress

Adulthood is biologically defined as the age at which sexual maturity is reached. This holds true for rodents or other animals, but in humans, adulthood is associated with several psychological and cultural concepts [64]. According to the World Health Organization (WHO), “an adult is a person older than 19 years of age unless national law delimits an earlier age” [82]. As mice reach sexual maturity at 8–12 weeks of age, mice older than 8 weeks of age are considered adult [64].

It is well known that stress, a prevalent experience in modern society, is a major predisposing and triggering factor for mood disorders in humans. Patients suffering from different psychiatric and mental disorders, such as major depression, anxiety disorder or post-traumatic stress disorder (PTSD), often show functional abnormalities in limbic structures, including the amygdala [104,105].

Rodent models of adult stress (Figure 1) not only can recapitulate these abnormalities observed in patients, but can trigger the emergence of symptoms resembling those of human psychiatric disorders [106]. The great majority of the preclinical research on stress has focused for many years on its effects during adulthood, so it is not surprising that the majority of our knowledge concerning the impact of stress on the amygdala and socio-affective behavior derives from adult stress studies. Since many reviews on this topic are available [13,14,16,30,107,108], we will just provide a summary of the principal consequences of adult stressors for the neuroarchitecture and function of the amygdala (Table 4).

Several chronic stress procedures have been used in rodents to model the behavioral and neuroanatomical consequences of maladaptive-stress exposure in humans, seeking to achieve a measure of construct validity. The three most commonly employed models are chronic restraint stress (CRS), chronic unpredictable stress (CUS) and chronic social defeat stress (CSDS). All these models elicit depressive-like behavior and alterations in sociability [108]. Other models are based in artificially disrupting the animal’s glucocorticoid homeostasis (i.e., chronic glucocorticoid treatment, genetic mutant mice expressing abnormal levels of glucocorticoid receptors in the brain) [108].

In a recent review, Dr. Qiao and colleagues extensively discussed the effects of adult stress on the dendrites and spines of different brain regions, including the amygdala [107]. As a summary of their findings, one can say that rodent studies showed that chronic stress suffered during adulthood generally resulted in an increased spine density and dendritic arborization in the amygdala (Figure 2). Specifically, CRS caused a decrease in the spine density of spiny neurons in the MeA nucleus of the amygdala, which are GABAergic neurons [107,109]. By contrast, CUS caused an increase in dendritic arborization and spine density in the BLA spiny neurons, which are glutamatergic neurons [107,115]. Interestingly, differently to hippocampal neurons, the dendritic hypertrophy described in spiny neurons from the BLA induced by CRS failed to be reversed after the same time period of stress-free recovery [107]. Moreover, although CRS showed no structural effect on BLA stellate neurons, a dendritic shrinkage was observed in MeA stellate neurons, together with a reduced social interaction and high vulnerability to social defeat stress [110]. Interestingly, it has also been demonstrated that, by modulating the duration of restraint stress in adult rodents, it is possible to induce the formation of new spines without remodeling dendrites in the BLA [120]. In the same way, repeated restraint stress (7 days) in adult rats increases the firing rate of BLA projecting neurons and increases anxiety-like behavior [111]. Similar to chronic stress protocols, both the acute (1 day) and chronic (10 days) exposure of mice to exogenous corticosterone increases dendritic length and spine density in the BLA. These changes were correlated with increased anxiety-like behavior after chronic or acute corticosterone treatment [119].

As we just mentioned, some studies have revealed dramatic alterations in the connectivity and structure of excitatory neurons in the amygdala after chronic stress in rodents [107]. However, chronic stress also induces changes in the structure and connectivity of interneurons in the amygdala [112,113]. At least some of these alterations seem to be facilitated by plasticity-related molecules associated to interneurons, such as the polysialylated form of the neural cell adhesion molecule (PSA-NCAM). Mice subjected to CRS (21 days) displayed decreased GAD67, synatophysin and PSA-NCAM protein levels in the amygdala and reduced dendritic arborization of interneurons in the BLA [112]. However, when a short CRS protocol (10 days) was applied to rats, an increased number of parvalbumin-expressing neurons was detected [113]. These findings were in line with results from the postmortem amygdala biopsies of major depression patients, which showed alterations in several synaptic and plasticity-related molecules. Specifically, researchers found decreased PSA-NCAM protein levels in the BLA and BM, decreased synaptophysin in the LA and BLA, and decreased GAD67 protein levels in the BMA, while the VGLUT-1 protein level was increased in the LA of depressed patients [121]. The unpredictable chronic mild stress protocol (UCMS) in mice has also been demonstrated to induce alterations in plasticity and synaptic strengthening in the amygdala [116]. Specifically, UCMS-induced elevated behavioral emotionality was found to correlate with the enlarged volume of the amygdala and an increased postsynaptic density-95 protein level [116].

Chronic stress exposure during adulthood alters the eCB system in many regions involved in emotional processing, including the amygdala, as we have already discussed in previous sections for other age periods. Specifically, exposure to CRS downregulated CB1-R expression in the amygdala of mice. It also increased fatty acid amide hydrolase (FAAH) activity and decreased the amount of the eCB N-arachidonylethanolamine (AEA) within the amygdala, which would be expected to decrease eCB signaling at the level of ligand availability [114] (Figure 3). In the same study, authors reported increased anxiety-like behavior in CRS mice, together with increased dendritic complexity, arborization and spine density of the pyramidal neurons in the BLA [114].

The activation of the HPA axis could be studied by analyzing the altered expression of some genes, such as vasopressin and proopiomelanocortin (POMC), after stress exposure [122,123]. Indeed, anxiety-related behaviors and aggression are regulated by brain vasopressin and oxytocin [124]. Chronic defeat stress increased the expression of POMC in the amygdala of adult mice [117]. In the same way, chronic social instability stress increased amygdaloid expression of POMC and OXTR in the amygdala and reduced the expression of AVPR1a in the same region. These changes were correlated with increased anxiety behavior after the stress protocol [118].

## 6. Stress Exposure during Old Age: Elderly Stress

Old age is biologically defined as the age of a particular individual who reaches or surpasses the average lifespan of his species. In humans, old age is commonly defined as having a chronological age of 65 years or older [125]. To define old age in rodents, senescence biomarkers must be significantly identified, and this usually occurs from 18 months of age onwards [64].

Senescence has long been viewed as a period of decreased adaptiveness to stress, in part due to the fact that depressive symptoms are very common in older people, although healthy aging has been associated with a stable emotional state and weakened brain responses to negative stimuli [126,127,128]. In a study comparing the effects of acute stress on reactions to happy and fearful facial expressions between old age (aged 60–75 years) and young adulthood (aged 18–30 years) people, researchers found that acute stress impaired emotional processing in healthy aged people, which may in turn increase their vulnerability to affective disorders. Specifically, they found that, although the physiological and affective responses to the stressor were very similar between the two age groups, only elderly subjects showed a stress-related increase in the neural activity of the amygdala [128].

One of the most well-known hypotheses that has been formulated to explain the relation between aging and the response to stress is the so-called “glucocorticoid cascade hypothesis of aging” [126,127]. In agreement with this hypothesis, it has been demonstrated that, although aged rats were capable of appropriately initiating a corticosterone stress response after immobilization stress (Figure 1), their capacity to finish it was dramatically impaired. When old and young-adult rats were monitored during the recovery period after immobilization, the corticosterone concentrations in young rats returned to basal range within 60 min after the end of stress, but the corticosterone concentrations in aged rats remained elevated for 24 h post stress, due to continued secretion of the hormone [126]. This problem of corticosterone hypersecretion has been suggested to result from degenerative changes within the aging brain, specifically in the limbic system [13]. In fact, many studies in non-stressed rodents have demonstrated an age-related hypertrophy of the amygdala [13]. Specifically, increased dendritic branching, neuronal loss and decreased synaptic afferences could be found in the BLA of non-stressed old-aged rats when compared to non-stressed adult rats [129]. Interestingly, this “normal” hypertrophy of the BLA during aging has been related to age-associated changes in the stress response, including decreases in corticotrophin-releasing factor (CRF)-binding proteins in the BLA of aged rats. Thus, old-aged BLA neurons become more vulnerable to dendritic hypertrophy caused by stress due to a decreased capacity to regulate CRF levels [130].

However, despite the importance and high prevalence of socio-affective-related disorders among elderly people, and the apparent role of the amygdala in these disorders, preclinical studies addressing these questions are still scarce.

In a study comparing stress-induced behavioral changes in mice exposed to mild social defeat stress, researchers found that mice that were stressed during old age (24-month-old) exhibited similar reduced social interactions (social avoidance behavior) to mice that were stressed during adulthood (8–16-week-old) when compared to matched non-stressed control mice. However, only the old-aged stressed group showed a decreased preference towards sucrose and an attenuated defeat-induced increase in water intake. By contrast, the young stressed and control groups did not display such anhedonic behavior. Interestingly, these findings reveal that the positive stimuli of hedonic behavior in aged mice becomes more vulnerable to social defeat [131].

Finally, in a study applying an acute restraint stress protocol that lasted 3 h, researchers found that young-adult (3-month-old) and aged rats (21-month-old) displayed equivalent levels of distress, as well as higher but equivalent glucocorticoid blood levels 21 h after restraint. However, aged but not young rats proved to be less responsive to new-onset acute stress, which may negatively impact long-term stress adaptation [132].

## 7. Conclusions

As a process orchestrated by the brain, the stress response varies across the lifespan. The studies in humans and animal models reviewed here reveal that, while deficits in sociability and social interaction/communication are shared consequences of stress exposure across all stages of life, depression- and anxiety-like symptoms are more variable among age periods, and aggression is just documented as a consequence of peripubertal period. Early exposure to stressful events (prenatal, perinatal, peripubertal) can trigger immediate molecular and cellular changes in the amygdala, and thus re-shape the way the brain reacts to stress in adulthood towards maladaptive responses. However, although stress suffered during early life can have dramatic consequences, such as the development of psychopathologies and pathological behavior later in life, it has also been demonstrated to be reversible when environmental enrichment or antidepressant treatment is administered early. Maladaptive responses can also be triggered by exposure to stress later in life, during adulthood or even old age. Particularly interesting is the stress response during old age; while elderly subjects are able to equally respond to a chronic or acute stressor, when a second stressor is presented, they show hypo-responsiveness, making them more vulnerable to the stressor, which may lead to psychopathologies. Regarding the molecular and cellular consequences of stress in the amygdala, the studies reviewed here show alterations in plasticity, eCB system and neuronal cytoarchitecture in all stages of life, suggesting that the amygdala remains vulnerable to stress throughout the whole of life, and therefore remains susceptible to being re-shaped.

## Figures and Tables

**Table 1:** Summary of the main molecular, cellular and behavioral alterations reported after prenatal stress exposure in rodents.

Stressor	Stress Protocol	Molecular/Cellular		Behavior		References
		Assessment	Age	Assessment	Age	
Restraint or immobilization	From G14 to G21 (45 min; 3 times/day)	↑ increased dendritic length of pyramidal and stellate neurons (BLA and LA)	P52			[44]
	From G10 to G16 (2 h/day)	↓ serotonergic metabolism, ↑ CORT response to social interaction	P70	↓ social interaction	P60 to P70	[45]
	3 days (2 h/day) + tail-shocks	↓ CB1-R in the amygdala			P60	[46]
Continuous light exposure	From G12 to G21	↑ RORA in the amygdala	P72	↓ mobility and exploration	P60 to P72	[47]
Unpredictable stress	Constant light exposure, fox-odor, restraint, cage changing, noise, wet bedding (from G6 to G21 in 5-HTT heterozygous mice)			↓ social interaction,↓ social interest	P60 to P62	[48]
	Restraint, social defeat, cold exposure, forced swim, open field, lights-on overnight (from G14 to G21)	↓ Bdnf expression, ↑ DNA methylation Bdnf exon IV, ↑ DNA methyltransferases in the amygdala	P21, P80			[49]
	Foot shocks (from G17 to G20)	↓ excitability of BLA pyramidal neurons	P10, P14, P17, P21, P28, P60	↓ sociability, ↓ anxiety	P7, P17, P60	[50]
	cage change and saline s.c. injection (from G14 to G21)	↓ number of neurons and glia in BLA and CeA	P7, P45 P60			[51]
	Restraint, social defeat, cold exposure, forced swim, open field, lights-on overnight (from G14 to G21)	↑ oxytocin receptor (OXTR) binding in the CeA		↓ social interaction, = anxiety	P56 to P64	[52]
Glucocorticoid administration	dexamethasone injections (0.4 mg/kg from G14 to G17 or 1 mg/kg from G18 to G19)	↓ number of CR+ interneurons in the LA	P60	depressive-like behavior, ↓ social interaction	P39, P50, P90, P120	[53,54,55]

Symbols and abbreviations: ↑ (increase), ↓ (decrease), = (no change), G (gestational day), P (postnatal day), BLA (basolateral nuclei of the amygdala), LA (lateral nuclei of the amygdala), CORT (corticosterone), CB1-R (cannabinoid receptor 1), RORA (circadian rhythm-related gene: RAR related orphan receptor A), 5-HTT (serotonin transporter), Bdnf (brain derived neurotrophic factor, gene), BLA (basolateral nucleus of the amygdala), CeA (central nucleus of the amygdala), s.c. (subcutaneous injection).

**Table 2:** Summary of the main molecular, cellular and behavioral alterations reported after perinatal stress exposure in rodents.

Stressor	Stress Protocol	Molecular/Cellular		Behavior		References
		Assessment	Age	Assessment	Age	
Bad caregiving conditions	Limited bedding (P1–P9) + 60 min immobilization (P10) or restraint (P20)	↑ spine density, ↑ spine number and ↑ excitability (pyramidal neurons BLA) ↑ BLA volume	P10, P20, P18 to P22	↑ anxiety ↓ social contact	P60 to P78	[66]
	Caregiver maltreatment (stressed dam) outside the home cage (30 min/day from P1 to P7)	↑ DNA methylation Bdnf exon IV (amygdala females) ↓ 5-hmC levels in (amygdala males)	P8, P30, P90	↑anxiety	P1 to P7	[67,68,69]
	Insufficient bedding (from P8 to P12)	↑ amygdala neuronal activity	P20, P45	↓ social behavior, depressive-like symptoms	P20, P45	[70]
Maternal separation	3 h/day from P2 to P12 in the home cage (dam removed)	↓ CB1-R binding sites in the amygdala (P40, P70), ↓ AEA content (P12, P14), ↑ 2-AG content (P12, P14)	P2, P12, P14, P40, P70			[71,72]
	30 min/day on P5, P16 and P21 outside the home cage (pups removed)	↑ BDNF protein levels in the amygdala	adult	↓ anxiety	Infant, Adult	[73]
	180 min/day from P2 to P14 outside the home cage (pups removed)	↑ BDNF expression in the CeA ↑ TrkB expression in the CeA	adult			[74]
	3 h/day from P5 to P10 outside the home cage (pups removed)	↑ DNA methyltransferases in the amygdala (P31) ↓ BDNF in the amygdala (P31, P86)	P31, P86	↓ social interaction	P30 to P31, P85 to P86	[75]
	1 h/day from P1 to P21 outside the home cage (pups removed and placed individually)	↓ spine density in apical dendrites (pyramidal neurons MeA)	P21			[76]
Drug administration (Δ9-THC)	5 mg/kg (oral in mothers) from G5 to weaning age (P24)	↓ density μ-opioid-R in posteromedial cortical amygdala (males) ↑ density μ-opioid-R in posteromedial cortical amygdala (females)	P70			[77]

Symbols and abbreviations: ↑ (increase), ↓ (decrease), G (gestational day), P (postnatal day), BLA (basolateral nuclei of the amygdala), LA (lateral nuclei of the amygdala), CB1-R (cannabinoid receptor 1), Bdnf (brain derived neurotrophic factor, gene), BDNF (brain derived neurotrophic factor, protein), 5hmC (5-Hydroxymethylcytosine), BLA (basolateral nucleus of the amygdala), CeA (central nucleus of the amygdala), TrKB (tropomyosin receptor kinase B), Δ9-THC (Δ9 tetrahydrocannabinol), AEA (anandamide), 2-AG (2-arachidonoyl glycerol), MeA (medial nucleus of the amygdala).

**Table 3:** Summary of the main molecular, cellular and behavioral alterations reported after peripubertal stress exposure in rodents.

Stressor	Stress Protocol	Molecular/Cellular		Behavior		References
		Assessment	Age	Assessment	Age	
Post-weaning social isolation stress model (PWSI)	Individual cages from P21 to P82	↓ BDNF expression (amygdala)	P82	pathological aggression, ↓ social communication	P82	[9,94]
	Individual cages from P28 to P109	↑ BLA pyramidal cell excitability	P101–P115	↑ anxiety	P101–P115	[95]
	Individual cages from P21 to P90	↑ GAD67 protein (CeM, MeA, BLA)	P90			[96]
	Individual cages from P21 to P90	↑ BLA, BMA, Ce volume ↑ number of PV+ interneurons (BLA, BMA) ↓ PSA-NCAM protein (amygdala) ↓ VGLUT1/VGAT (LA, BLA) ↑ mRNA CB1-R (amygdala)	P90	↑ anxiety	P90	[97]
Unpredictable stress	fear-inducing stressors: open field, fox-odor, elevated platform (P28–P30, P34, P36, P40, P42)	↑ mRNA NR1 (amygdala) ↓ mRNA GAD67 (amygdala) ↑ VGLUT1/VGAT (CeA) ↑ mRNA encoding GR and ↓ number of GR + cells (CeA) ↓ GAD and GABA-A receptor α3 (LA, BLA, BMA, MeA, CeA)	P90	↓ sociability, ↑ anxiety, ↑ novelty reactivity, pathological aggression	P90	[83,98,99,100,101]
Repeated restraint stress	20 min/day from P29 to P37	↓ GABAergic inhibition of LA projection neurons ↓ presynaptic GABA function and interneuron activity (LA)	P39	↑ anxiety	P38	[102]
	2 h/day restraint session + 40 tail shocks /day (from P22 to P24)	↓ serotoninergic modulation of GABAergic transmission (BLA), amygdala hyperexcitability	P24–P25			[103]

Symbols and abbreviations: ↑ (increase), ↓ (decrease), P (postnatal day), BLA (basolateral nuclei of the amygdala), BMA (basomedial nucleus of the amygdala), LA (lateral nuclei of the amygdala), MeA (medial nucleus of the amygdala), CeA (central nucleus of the amygdala), CB1-R (cannabinoid receptor 1), Bdnf (brain derived neurotrophic factor, gene), BDNF (brain derived neurotrophic factor, protein), GAD (glutamic acid decarboxylase), GABA (gamma-amino butyric acid), GABA-A (GABA type A receptor), NR1 (subunit 1 of the N-methyl-D-aspartate-receptor), GR (glucocorticoid receptor), VGLUT (vesicular glutamate transporter), VGAT (vesicular GABA transporter).

**Table 4:** Summary of the main molecular, cellular and behavioral alterations reported after adult stress exposure in rodents.

Stressor	Stress Protocol	Molecular/Cellular		Behavior		References
		Assessment	Age	Assessment	Age	
CRS	6 h/day for 21 days	↓ spine density of spiny neurons in the MeA (GABAergic)	P90	depression-like behavior	P90	[107,109]
	2 h/day for 21 days	Dendritic shrinkage in MeA stellate neurons (↓ arborization, ↓ dendrite length)	P77	↓ social interaction ↑ anxiety-like behavior	P77	[110]
	1 h/day for 7 days	↑ firing rate of BLA projecting neurons	P65	↑ anxiety-like behavior	P64	[111]
	1 h/day for 21 days	↓ GAD67, synaptophysin and PSA-NCAM in the amygdala ↓ dendritic arborization of interneurons in the BLA	P112			[112]
	6 h/day for 10 days	↑ number of PV + neurons in the BLA	P112			[113]
	6 h/day for 21 days	↓ CB1-R expression in the amygdala ↑ FAAH activity, ↓ AEA amygdala ↑ dendritic arborization, complexity and spine density of pyramidal neurons in the BLA	P112	↑ anxiety-like behavior	P112	[114]
CUS (Forced swim, restraint, lights-on overnight, aversive smell, wet bedding, no bedding)	2 weeks	↑ dendritic arborization and spine density in the BLA spiny neurons (glutamatergic neurons)	P98	depression-like behavior	P98	[107,115]
	5 weeks	↑ postsynaptic density-95 protein level in the amygdala and synaptic strengthening	P90	↑ behavioral emotionality	P90	[116]
Chronic social stress	Chronic defeat stress (5 min/day for 5 days)	↑ POMC in the amygdala	P77			[117]
	Unpredictable chronic social instability (isolation and crowding, 3 h or 6 h/day for 28 days)	↑ POMC, ↑ OXTR and ↓ AVPR1a in the amygdala	P88	↑ anxiety-like behavior	P88	[118]
Chronic exposure to exogenous corticosterone	10 mg/kg (s.c.) for 1 or 10 days	↑ dendritic length ↑ spine density (pyramidal neurons BLA)	P85	↑ anxiety-like behavior	P85	[119]

Symbols and abbreviations: ↑ (increase), ↓ (decrease), P (postnatal day), BLA (basolateral nuclei of the amygdala), BMA (basomedial nucleus of the amygdala), LA (lateral nuclei of the amygdala), MeA (medial nucleus of the amygdala), CeA (central nucleus of the amygdala), CB1-R (cannabinoid receptor 1), FAAH (fatty acid amine hydrolase), POMC (proopiomelanocortin), AEA (anandamide), OXTR (oxytocin receptor), AVPR1a (arginine vasopressin receptor 1a), GAD 67 (glutamic acid decarboxylase enzyme, 67 kDa), GABA (gamma-amino butyric acid), PSA-NCAM (polysialylated form of the neural cell adhesion molecule), PV (parvalbumin), s.c. (subcutaneous injection).

## Abbreviations

AA	Arachidonic acid
ACTH	Adrenocorticotropic hormone
AEA	Anandamide
ASD	Autism spectrum disorders
Bdnf	Brain derived neurotrophic factor
BMA	Basomedial nucleus of the amygdala
BLA	Basolateral nucleus of the amygdala
CB1-R	Cannabinoid type 1 receptor
CeA	Central nucleus of the amygdala
CeM	Centromedial nucleus of the amygdala
CORT	Corticosterone
CRS	Chronic restraint stress
CRF	Corticotrophin releasing factor
CSDS	Chronic social defeat stress
CUS	Chronic unpredictable stress
Dnmt	DNA methyltransferase
eCB	Endocannabinoid
FAAH	Fatty acid amine hydrolase
GAD67	Glutamic acid decarboxylase enzime, 67KDa
GR	Gucocorticoid receptor
HPA	Hypothalamic pituitary adrenal
LA	Lateral nucleus of the amygdala
MeA	Medial nucleus of the amygdala
NR-1	Subunit 1 of the N-Metyl-D-Aspartate (NMDA) receptor
OXTR	Oxytocin receptor
POMC	Proopiomelano-cortin
PSA-NCAM	Polysialylated form of the neural cell adhesion molecule (NCAM)
PTSD	Post-traumatic stress disorder
PV	Parvalbumin
PWSI	Post-weaning social isolation
RORA	RAR related orphan receptor A (circadian rhythm-related gene)
UCMS	Unpredictable chronic mild stress
VGAT	Vesicular GABA transporter
VGLUT1	Vesicular glutamate transporter type 1
WHO	World Health Organization
2-AG	2-arachidonoyl-glycerol
Δ9-THC	Δ9-Tetrahydrocannabinol

## References

[1] Young S.N. (2008). The neurobiology of human social behaviour: An important but neglected topic. J. Psychiatry Neurosci..

[2] Kennedy D.P., Adolphs R. (2012). The social brain in psychiatric and neurological disorders. Trends Cogn. Sci..

[3] Lee N.S., Beery A.K. (2019). Neural Circuits Underlying Rodent Sociality: A Comparative Approach. Current Topics in Behavioral Neurosciences.

[4] Insel T.R., Fernald R.D. (2004). How the brain processes social information: Searching for the social brain. Annu. Rev. Neurosci..

[5] Tzanoulinou S., Sandi C. (2015). The Programming of the Social Brain by Stress During Childhood and Adolescence: From Rodents to Humans. Social Behavior from Rodents to Humans.

[6] Hensch T.K., Bilimoria P.M. (2012). Re-opening Windows: Manipulating Critical Periods for Brain Development. Cerebrum.

[7] Marco E.M., MacRì S., Laviola G. (2011). Critical age windows for neurodevelopmental psychiatric disorders: Evidence from animal models. Neurotox. Res..

[8] Tóth M., Halász J., Mikics É., Barsy B., Haller J. (2008). Early Social Deprivation Induces Disturbed Social Communication and Violent Aggression in Adulthood. Behav. Neurosci..

[9] Toth M., Mikics E., Tulogdi A., Aliczki M., Haller J. (2011). Post-weaning social isolation induces abnormal forms of aggression in conjunction with increased glucocorticoid and autonomic stress responses. Horm. Behav..

[10] Sandi C., Haller J. (2015). Stress and the Social Brain: Behavioural Effects and Neurobiological Mechanisms. Nat. Rev. Neurosci..

[11] Paus T., Keshavan M., Giedd J.N. (2008). Why Do Many Psychiatric Disorders Emerge During Adolescence?. Nat. Rev. Neurosci..

[12] Bale T.L., Epperson C.N. (2015). Sex differences and stress across the lifespan. Nat. Neurosci..

[13] Novais A., Monteiro S., Roque S., Correia-Neves M., Sousa N. (2017). How age, sex and genotype shape the stress response. Neurobiol. Stress.

[14] Sousa N., Almeida O.F.X. (2012). Disconnection and reconnection: The morphological basis of (mal)adaptation to stress. Trends Neurosci..

[15] Beery A.K., Kaufer D. (2015). Stress, social behavior, and resilience: Insights from rodents. Neurobiol. Stress.

[16] McEwen B.S. (2017). Neurobiological and Systemic Effects of Chronic Stress. Chronic Stress.

[17] Yehuda R., Schmeidler J., Wainberg M., Binder-Brynes K., Duvdevani T. (1998). Vulnerability to Posttraumatic Stress Disorder in Adult Offspring of Holocaust Survivors. Am. J. Psychiatry.

[18] Franklin T., Russig H., Weiss I., Gräff J., Linder N., Michalon A., Vizi S., Mansuy I. (2010). Epigenetic Transmission of the Impact of Early Stress Across Generations. Biol. Psychiatry.

[19] Matthews S., Phillips D. (2012). Transgenerational Inheritance of Stress Pathology. Exp. Neurol..

[20] Hillis S., Mercy J., Amobi A., Kress H. (2016). Global prevalence of past-year violence against children: A systematic review and minimum estimates. Pediatrics.

[21] Stöckl H., March L., Pallitto C., Garcia-Moreno C. (2014). Intimate partner violence among adolescents and young women: Prevalence and associated factors in nine countries: A cross-sectional study. BMC Public Health.

[22] Burkle F., Argent A., Kissoon N. (2011). The Reality of Pediatric Emergency Mass Critical Care in the Developing World. Pediatr. Crit. Care Med..

[23] Wilkinson R. (2004). Why Is Violence More Common Where Inequality Is Greater?. Ann. N. Y. Acad. Sci..

[24] De Boer S.F. (2018). Animal models of excessive aggression: Implications for human aggression and violence. Curr. Opin. Psychol..

[25] Chen P., Hong W. (2018). Neural Circuit Mechanisms of Social Behavior. Neuron.

[26] Tottenham N., Sheridan M.A. (2010). A review of adversity, the amygdala and the hippocampus: A consideration of developmental timing. Front. Hum. Neurosci..

[27] Wellman L.L., Forcelli P.A., Aguilar B.L., Malkova L. (2016). Bidirectional control of social behavior by activity within basolateral and central amygdala of primates. J. Neurosci..

[28] Adolphs R. (2010). What does the amygdala contribute to social cognition?. Ann. N. Y. Acad. Sci..

[29] McEwen B.S., Bowles N.P., Gray J.D., Hill M.N., Hunter R.G., Karatsoreos I.N., Nasca C. (2015). Mechanisms of stress in the brain. Nat. Neurosci..

[30] McEwen B.S., Nasca C., Gray J.D. (2016). Stress Effects on Neuronal Structure: Hippocampus, Amygdala, and Prefrontal Cortex. Neuropsychopharmacology.

[31] Charil A., Laplante D.P., Vaillancourt C., King S. (2010). Prenatal stress and brain development. Brain Res. Rev..

[32] Coussons-Read M.E. (2013). Effects of prenatal stress on pregnancy and human development: Mechanisms and pathways. Obstet. Med..

[33] Huizink A.C., De Rooij S.R. (2018). Prenatal stress and models explaining risk for psychopathology revisited: Generic vulnerability and divergent pathways. Dev. Psychopathol..

[34] Weinstock M. (2017). Prenatal stressors in rodents: Effects on behavior. Neurobiol. Stress.

[35] King S., Dancause K., Turcotte-Tremblay A.-M., Veru F., Laplante D.P. (2012). Using natural disasters to study the effects of prenatal maternal stress on child health and development. Birth Defects Res. C Embryo Today.

[36] Lipner E., Murphy S.K., Ellman L.M. (2019). Prenatal Maternal Stress and the Cascade of Risk to Schizophrenia Spectrum Disorders in Offspring. Curr. Psychiatry Rep..

[37] Ellman L.M., Murphy S.K., Maxwell S.D., Calvo E.M., Cooper T., Schaefer C.A., Bresnahan M.A., Susser E.S., Brown A.S. (2019). Maternal cortisol during pregnancy and offspring schizophrenia: Influence of fetal sex and timing of exposure. Schizophr. Res..

[38] Levine S.Z., Levav I., Goldberg Y., Pugachova I., Becher Y., Yoffe R. (2016). Exposure to genocide and the risk of schizophrenia: A population-based study. Psychol. Med..

[39] Beversdorf D.Q., Stevens H.E., Margolis K.G., Van de Water J. (2019). Prenatal Stress and Maternal Immune Dysregulation in Autism Spectrum Disorders: Potential Points for Intervention. Curr. Pharm. Des..

[40] Walder D.J., Laplante D.P., Sousa-Pires A., Veru F., Brunet A., King S. (2014). Prenatal maternal stress predicts autism traits in 61/2 year-old children: Project Ice Storm. Psychiatry Res..

[41] Van Den Bergh B.R.H., Van Calster B., Smits T., Van Huffel S., Lagae L. (2008). Antenatal maternal anxiety is related to HPA-axis dysregulation and self-reported depressive symptoms in adolescence: A prospective study on the fetal origins of depressed mood. Neuropsychopharmacology.

[42] Thomason M.E. (2020). Development of Brain Networks In Utero: Relevance for Common Neural Disorders. Biol. Psychiatry.

[43] Scheinost D., Kwon S.H., Lacadie C., Sze G., Sinha R., Constable R.T., Ment L.R. (2016). Prenatal stress alters amygdala functional connectivity in preterm neonates. NeuroImage Clin..

[44] Ancatén González C., Gutiérrez-Rojas C., Bustamante Valdés C. (2017). Maternal exercise reverses morphologic changes in amygdala neurons produced by prenatal stress. Neurol. Psychiatry Brain Res..

[45] Gur T.L., Palkar A.V., Rajasekera T., Allen J., Niraula A., Godbout J., Bailey M.T. (2019). Prenatal stress disrupts social behavior, cortical neurobiology and commensal microbes in adult male offspring. Behav. Brain Res..

[46] Xing G., Carlton J., Jiang X., Wen J., Jia M., Li H. (2014). Differential Expression of Brain Cannabinoid Receptors between Repeatedly Stressed Males and Females may Play a Role in Age and Gender-Related Difference in Traumatic Brain Injury: Implications from Animal Studies. Front. Neurol..

[47] Voiculescu S.E., Le Duc D., Roșca A.E., Zeca V., Chiţimuș D.M., Arsene A.L., Drăgoi C.M., Nicolae A.C., Zăgrean L., Schöneberg T. (2016). Behavioral and molecular effects of prenatal continuous light exposure in the adult rat. Brain Res..

[48] Jones K.L., Smith R.M., Edwards K.S., Givens B., Tilley M.R., Beversdorf D.Q. (2010). Combined effect of maternal serotonin transporter genotype and prenatal stress in modulating offspring social interaction in mice. Int. J. Dev. Neurosci..

[49] Boersma G.J., Lee R.S., Cordner Z.A., Ewald E.R., Purcell R.H., Moghadam A.A., Tamashiro K.L. (2014). Prenatal stress decreases Bdnf expression and increases methylation of Bdnf exon IV in rats. Epigenetics.

[50] Ehrlich D.E., Rainnie D.G. (2015). Prenatal Stress Alters the Development of Socioemotional Behavior and Amygdala Neuron Excitability in Rats. Neuropsychopharmacology.

[51] Kraszpulski M., Dickerson P.A., Salm A.K. (2006). Prenatal stress affects the developmental trajectory of the rat amygdala. Stress.

[52] Lee P.R., Brady D.L., Shapiro R.A., Dorsa D.M., Koenig J.I. (2007). Prenatal stress generates deficits in rat social behavior: Reversal by oxytocin. Brain Res..

[53] Roque S., Oliveira T.G., Nobrega C., Barreira-Silva P., Nunes-Alves C., Sousa N., Palha J.A., Correia-Neves M. (2011). Interplay between Depressive-Like Behavior and the Immune System in an Animal Model of Prenatal Dexamethasone Administration. Front. Behav. Neurosci..

[54] Borges S., Coimbra B., Soares-Cunha C., Miguel Pêgo J., Sousa N., João Rodrigues A. (2013). Dopaminergic modulation of affective and social deficits induced by prenatal glucocorticoid exposure. Neuropsychopharmacology.

[55] Zuloaga D.G., Carbone D.L., Handa R.J. (2012). Prenatal dexamethasone selectively decreases calretinin expression in the adult female lateral amygdala. Neurosci. Lett..

[56] Won J., Jin Y., Choi J., Park S., Lee T.H., Lee S.-R., Chang K.-T., Hong Y. (2017). Melatonin as a Novel Interventional Candidate for Fragile X Syndrome with Autism Spectrum Disorder in Humans. Int. J. Mol. Sci..

[57] A Quera Salva M., Hartley S., Barbot F., C Alvarez J., Lofaso F., Guilleminault C. (2011). Circadian Rhythms, Melatonin and Depression. Curr. Pharm. Des..

[58] Ehrlich D.E., Neigh G.N., Bourke C.H., Nemeth C.L., Hazra R., Ryan S.J., Rowson S., Jairam N., Sholar C.A., Rainnie D.G. (2015). Prenatal stress, regardless of concurrent escitalopram treatment, alters behavior and amygdala gene expression of adolescent female rats. Neuropharmacology.

[59] Salari A.-A., Fatehi-Gharehlar L., Motayagheni N., Homberg J.R. (2016). Fluoxetine normalizes the effects of prenatal maternal stress on depression- and anxiety-like behaviors in mouse dams and male offspring. Behav. Brain Res..

[60] Pilkay S.R., Combs-Orme T., Tylavsky F., Bush N., Smith A.K. (2020). Maternal trauma and fear history predict BDNF methylation and gene expression in newborns. PeerJ.

[61] Franks A., Berry K., DeFranco D. (2020). Prenatal Drug Exposure and Neurodevelopmental Programming of Glucocorticoid Signalling. J. Neuroendocrinol..

[62] Dow-Edwards D., Frank A., Wade D., Weedon J., Izenwasser S. (2016). Sexually-dimorphic alterations in cannabinoid receptor density depend upon prenatal/early postnatal history. Neurotoxicol. Teratol..

[63] Morena M., Patel S., Bains J.S., Hill M.N. (2016). Neurobiological Interactions Between Stress and the Endocannabinoid System. Neuropsychopharmacology.

[64] Dutta S., Sengupta P. (2016). Men and mice: Relating their ages. Life Sci..

[65] Huppertz-Kessler C.J., Poeschl J., Hertel R., Unsicker K., Schenkel J. (2012). Effects of a new postnatal stress model on monoaminergic neurotransmitters in rat brains. Brain Dev..

[66] Guadagno A., Wong T.P., Walker C.-D. (2018). Morphological and functional changes in the preweaning basolateral amygdala induced by early chronic stress associate with anxiety and fear behavior in adult male, but not female rats. Prog. Neuro-Psychopharmacol. Biol. Psychiatry.

[67] Szyf M. (2019). The epigenetics of perinatal stress. Dialogues Clin. Neurosci..

[68] Roth T.L., Matt S., Chen K., Blaze J. (2014). Bdnf DNA methylation modifications in the hippocampus and amygdala of male and female rats exposed to different caregiving environments outside the homecage. Dev. Psychobiol..

[69] Doherty T.S., Forster A., Roth T.L. (2016). Global and gene-specific DNA methylation alterations in the adolescent amygdala and hippocampus in an animal model of caregiver maltreatment. Behav. Brain Res..

[70] Raineki C., Cortés M.R., Belnoue L., Sullivan R.M. (2012). Effects of early-life abuse differ across development: Infant social behavior deficits are followed by adolescent depressive-like behaviors mediated by the amygdala. J. Neurosci..

[71] Goldstein Ferber S., Trezza V., Weller A. (2019). Early life stress and development of the endocannabinoid system: A bidirectional process in programming future coping. Dev. Psychobiol..

[72] Hill M.N., Eiland L., Lee T.T., Hillard C.J., McEwen B.S. (2019). Early Life Stress Alters the Developmental Trajectory of Corticolimbic Endocannabinoid Signaling in Male Rats. Neuropharmacology.

[73] Berman A.K., Lott R.B., Donaldson S.T. (2014). Periodic maternal deprivation may modulate offspring anxiety-like behavior through mechanisms involving neuroplasticity in the amygdala. Brain Res. Bull..

[74] Chung E.K.Y., Bian Z.X., Xu H.X., Sung J.J.Y. (2009). Neonatal maternal separation increases brain-derived neurotrophic factor and tyrosine kinase receptor B expression in the descending pain modulatory system. NeuroSignals.

[75] Karen C., Rajan K.E. (2019). Social Behaviour and Epigenetic Status in Adolescent and Adult Rats: The Contribution of Early-Life Stressful Social Experience. Cell. Mol. Neurobiol..

[76] Poeggel G., Helmeke C., Abraham A., Schwabe T., Friedrich P., Braun K. (2003). Juvenile emotional experience alters synaptic composition in the rodent cortex, hippocampus, and lateral amygdala. Proc. Natl. Acad. Sci. USA.

[77] Vela G., Martín S., García-Gil L., Crespo J.A., Ruiz-Gayo M., Javier Fernández-Ruiz J., García-Lecumberri C., Pélaprat D., Fuentes J.A., Ramos J.A. (1998). Maternal exposure to δ9-tetrahydrocannabinol facilitates morphine self-administration behavior and changes regional binding to central μ opioid receptors in adult offspring female rats. Brain Res..

[78] Keller S.M., Nowak A., Roth T.L. (2019). Female pups receive more maltreatment from stressed dams. Dev. Psychobiol..

[79] Raineki C., Opendak M., Sarro E., Showler A., Bui K., McEwen B.S., Wilson D.A., Sullivan R.M. (2019). During infant maltreatment, stress targets hippocampus, but stress with mother present targets amygdala and social behavior. Proc. Natl. Acad. Sci. USA.

[80] Higuera-Matas A., Ucha M., Ambrosio E. (2015). Long-term consequences of perinatal and adolescent cannabinoid exposure on neural and psychological processes. Neurosci. Biobehav. Rev..

[81] Spear L.P. (2000). The adolescent brain and age-related behavioral manifestations. Neurosci. Biobehav. Rev..

[82] WHO (2013). Definition of Key Terms.

[83] Tzanoulinou S., Riccio O., De Boer M.W., Sandi C. (2014). Peripubertal stress-induced behavioral changes are associated with altered expression of genes involved in excitation and inhibition in the amygdale. Transl. Psychiatry.

[84] Stain H., Brønnick K., Hegelstad W.T., Joa I., Johannessen J., Langeveld J., Mawn L., Larsen T. (2014). Impact of Interpersonal Trauma on the Social Functioning of Adults With First-Episode Psychosis. Schizophr. Bull..

[85] Palmier-Claus J., Berry K., Darrell-Berry H., Emsley R., Parker S., Drake R., Bucci S. (2016). Childhood adversity and social functioning in psychosis: Exploring clinical and cognitive mediators. Psychiatry Res..

[86] Kilian S., Asmal L., Chiliza B., Olivier M., Phahladira L., Scheffler F., Seedat S., Marder S., Green M., Emsley R. (2018). Childhood adversity and cognitive function in schizophrenia spectrum disorders and healthy controls: Evidence for an association between neglect and social cognition. Psychol. Med..

[87] Heim C., Binder E. (2012). Current Research Trends in Early Life Stress and Depression: Review of Human Studies on Sensitive Periods, Gene-Environment Interactions, and Epigenetics. Exp. Neurol..

[88] Widom C.S., DuMont K., Czaja S.J. (2007). A prospective investigation of major depressive disorder and comorbidity in abused and neglected children grown up. Arch. Gen. Psychiatry.

[89] Widom C.S., Maxfield M.G. (1996). A prospective examination of risk for violence among abused and neglected children. Ann. N. Y. Acad. Sci..

[90] Yeager C.A., Lewis D.O. (2000). Mental illness, neuropsychologic deficits, child abuse, and violence. Child Adolesc. Psychiatr. Clin. N. Am..

[91] Van der Kolk B.A. (2003). The neurobiology of childhood trauma and abuse. Child Adolesc. Psychiatr. Clin. N. Am..

[92] Malvaso C., Day A., Casey S., Corrado R. (2017). Young Offenders, Maltreatment, and Trauma: A Pilot Study. Psychiatry Psychol. Law.

[93] Brydges N.M., Hall J., Best C., Rule L., Watkin H., Drake A.J., Lewis C., Thomas K.L., Hall J. (2019). Childhood stress impairs social function through AVP-dependent mechanisms. Transl. Psychiatry.

[94] Mikics É., Guirado R., Umemori J., Tóth M., Biró L., Miskolczi C., Balázsfi D., Zelena D., Castrén E., Haller J. (2018). Social learning requires plasticity enhanced by fluoxetine through prefrontal Bdnf-TrkB signaling to limit aggression induced by post-weaning social isolation. Neuropsychopharmacology.

[95] Rau A.R., Chappell A.M., Butler T.R., Ariwodola O.J., Weiner J.L. (2015). Increased Basolateral Amygdala Pyramidal Cell Excitability May Contribute to the Anxiogenic Phenotype Induced by Chronic Early-Life Stress. J. Neurosci..

[96] Gilabert-Juan J., Moltó M.D., Nacher J. (2012). Post-weaning social isolation rearing influences the expression of molecules related to inhibitory neurotransmission and structural plasticity in the amygdala of adult rats. Brain Res..

[97] Castillo-Gómez E., Pérez-Rando M., Bellés M., Gilabert-Juan J., Llorens J.V., Carceller H., Bueno-Fernández C., García-Mompó C., Ripoll-Martínez B., Curto Y. (2017). Early social isolation stress and perinatal nmda receptor antagonist treatment induce changes in the structure and neurochemistry of inhibitory neurons of the adult amygdala and prefrontal cortex. eNeuro.

[98] Márquez C., Poirier G., Cordero M., Larsen M., Groner A., Marquis J., Magistretti P., Trono D., Sandi C. (2013). Peripuberty Stress Leads to Abnormal Aggression, Altered Amygdala and Orbitofrontal Reactivity and Increased Prefrontal MAOA Gene Expression. Transl. Psychiatry.

[99] Cordero M.I., Poirier G.L., Marquez C., Veenit V., Fontana X., Salehi B., Ansermet F., Sandi C. (2012). Evidence for biological roots in the transgenerational transmission of intimate partner violence. Transl. Psychiatry.

[100] Tzanoulinou S., García-Mompó C., Castillo-Gómez E., Veenit V., Nacher J., Sandi C. (2014). Long-Term Behavioral Programming Induced by Peripuberty Stress in Rats Is Accompanied by GABAergic-Related Alterations in the Amygdala. PLoS ONE.

[101] Papilloud A., Veenit V., Tzanoulinou S., Riccio O., Zanoletti O., de Suduiraut I.G., Grosse J., Sandi C. (2019). Peripubertal stress-induced heightened aggression: Modulation of the glucocorticoid receptor in the central amygdala and normalization by mifepristone treatment. Neuropsychopharmacology.

[102] Zhang W., Rosenkranz J.A. (2016). Effects of Repeated Stress on Age-Dependent GABAergic Regulation of the Lateral Nucleus of the Amygdala. Neuropsychopharmacology.

[103] Jiang X., Xing G., Yang C., Verma A., Zhang L., Li H. (2009). Stress Impairs 5-HT2A Receptor-Mediated Serotonergic Facilitation of GABA Release in Juvenile Rat Basolateral Amygdala. Neuropsychopharmacology.

[104] Ferrari A.J., Somerville A.J., Baxter A.J., Norman R., Patten S.B., Vos T., Whiteford H.A. (2013). Global variation in the prevalence and incidence of major depressive disorder: A systematic review of the epidemiological literature. Psychol. Med..

[105] Tang S., Lu L., Zhang L., Hu X., Bu X., Li H., Hu X., Gao Y., Zeng Z., Gong Q. (2018). Abnormal amygdala resting-state functional connectivity in adults and adolescents with major depressive disorder: A comparative meta-analysis. EBioMedicine.

[106] Mothersill O., Donohoe G. (2016). Neural effects of social environmental stress—An activation likelihood estimation meta-analysis. Psychol. Med..

[107] Qiao H., Li M.-X., Xu C., Chen H.-B., An S.-C., Ma X.-M., Qiao H., Li M.-X., Xu C., Chen H.-B. (2016). Dendritic Spines in Depression: What We Learned from Animal Models. Neural Plast..

[108] Nestler E.J., Hyman S.E. (2010). Animal models of neuropsychiatric disorders. Nat. Neurosci..

[109] Bennur S., Shankaranarayana Rao B.S., Pawlak R., Strickland S., McEwen B.S., Chattarji S. (2007). Stress-induced spine loss in the medial amygdala is mediated by tissue-plasminogen activator. Neuroscience.

[110] Lau T., Bigio B., Zelli D., McEwen B., Nasca C. (2017). Stress-induced structural plasticity of medial amygdala stellate neurons and rapid prevention by a candidate antidepressant. Mol. Psychiatry.

[111] Zhang W., Rosenkranz J.A. (2012). Repeated restraint stress increases basolateral amygdala neuronal activity in an age-dependent manner. Neuroscience.

[112] Gilabert-Juan J., Castillo-Gomez E., Pérez-Rando M., Moltó M.D., Nacher J. (2011). Chronic stress induces changes in the structure of interneurons and in the expression of molecules related to neuronal structural plasticity and inhibitory neurotransmission in the amygdala of adult mice. Exp. Neurol..

[113] Pesarico A.P., Bueno-Fernandez C., Guirado R., Gómez-Climent M.Á., Curto Y., Carceller H., Nacher J. (2019). Chronic Stress Modulates Interneuronal Plasticity: Effects on PSA-NCAM and Perineuronal Nets in Cortical and Extracortical Regions. Front. Cell. Neurosci..

[114] Hill M.N., Kumar S.A., Filipski S.B., Iverson M., Stuhr K.L., Keith J.M., Cravatt B.F., Hillard C.J., Chattarji S., McEwen B.S. (2013). Disruption of fatty acid amide hydrolase activity prevents the effects of chronic stress on anxiety and amygdalar microstructure. Mol. Psychiatry.

[115] Sharma H.R., Thakur M.K. (2015). Correlation of ERα/ERβ expression with dendritic and behavioural changes in CUMS mice. Physiol. Behav..

[116] Nikolova Y.S., Misquitta K.A., Rocco B.R., Prevot T.D., Knodt A.R., Ellegood J., Voineskos A.N., Lerch J.P., Hariri A.R., Sibille E. (2018). Shifting priorities: Highly conserved behavioral and brain network adaptations to chronic stress across species. Transl. Psychiatry.

[117] Sachs B.D., Tran H.L., Folse E., Caron M.G. (2018). Brain-region-specific molecular responses to maternal separation and social defeat stress in mice. Neuroscience.

[118] Nowacka-Chmielewska M.M., Kasprowska-Liśkiewicz D., Barski J.J., Obuchowicz E., Małecki A. (2017). The behavioral and molecular evaluation of effects of social instability stress as a model of stress-related disorders in adult female rats. Stress.

[119] Mitra R., Sapolsky R.M. (2008). Acute corticosterone treatment is sufficient to induce anxiety and amygdaloid dendritic hypertrophy. Proc. Natl. Acad. Sci. USA.

[120] Mitra R., Jadhav S., McEwen B.S., Vyas A., Chattarji S. (2005). Stress duration modulates the spatiotemporal patterns of spine formation in the basolateral amygdala. Proc. Natl. Acad. Sci. USA.

[121] Varea E., Guirado R., Gilabert-Juan J., Martí U., Castillo-Gomez E., Blasco-Ibáñez J.M., Crespo C., Nacher J. (2012). Expression of PSA-NCAM and synaptic proteins in the amygdala of psychiatric disorder patients. J. Psychiatr. Res..

[122] Ancelin M.-L., Scali J., Norton J., Ritchie K., Dupuy A.-M., Chaudieu I., Ryan J. (2017). Heterogeneity in HPA axis dysregulation and serotonergic vulnerability to depression. Psychoneuroendocrinology.

[123] Du X., Pang T.Y. (2015). Is Dysregulation of the HPA-Axis a Core Pathophysiology Mediating Co-Morbid Depression in Neurodegenerative Diseases?. Front. Psychiatry.

[124] Lukas M., Neumann I. (2013). Oxytocin and Vasopressin in Rodent Behaviors Related to Social Dysfunctions in Autism Spectrum Disorders. Behav. Brain Res..

[125] WHO (2016). Proposed Working Definition of an Older Person in Africa for the MDS Project.

[126] Sapolsky R.M., Krey L.C., McEwen B.S. (1986). The neuroendocrinology of stress and aging: The glucocorticoid cascade hypothesis. Endocr. Rev..

[127] Mcewen B.S. (1992). Re-examination of the glucocorticoid hypothesis of stress and aging. Prog. Brain Res..

[128] Everaerd D., Klumpers F., Oude Voshaar R., Fernández G., Tendolkar I. (2017). Acute Stress Enhances Emotional Face Processing in the Aging Brain. Biol. Psychiatry Cogn. Neurosci. Neuroimaging.

[129] Rubinow M.J., Drogos L.L., Juraska J.M. (2009). Age-related dendritic hypertrophy and sexual dimorphism in rat basolateral amygdala. Neurobiol. Aging.

[130] Xiao C., Sartin J., Mulchahey J.J., Segar T., Sheriff S., Herman J.P., Kasckow J.W. (2006). Aging associated changes in amygdalar corticotropin-releasing hormone (CRH) and CRH-binding protein in Fischer 344 rats. Brain Res..

[131] Oizumi H., Kuriyama N., Imamura S., Tabuchi M., Omiya Y., Mizoguchi K., Kobayashi H. (2019). Influence of aging on the behavioral phenotypes of C57BL/6J mice after social defeat. PLoS ONE.

[132] Buechel H.M., Popovic J., Staggs K., Anderson K.L., Thibault O., Blalock E.M. (2014). Aged rats are hypo-responsive to acute restraint: Implications for psychosocial stress in aging. Front. Aging Neurosci..

